# Biomarker changes during long-term noninvasive ventilation in obesity hypoventilation syndrome: a prospective observational study

**DOI:** 10.1007/s11325-026-03785-x

**Published:** 2026-08-24

**Authors:** Luca Valko, Vivien Moro, Szabolcs Baglyas, Blanka Mezo, Eszter Podmaniczky, Zoltan Prohaszka, Andras Lorx

**Affiliations:** 1 Department of Anesthesiology and Intensive Therapy, Home Mechanical Ventilation Program, Semmelweis University, Budapest, Hungary; 2 HUN-REN-SE Research Group for Immunology and Hematology, Department of Internal Medicine and Hematology, Semmelweis University, Budapest, Hungary; 3 Department of Internal Medicine and Hematology, Semmelweis University, Budapest, Hungary

**Keywords:** Biomarker, Obesity hypoventilation syndrome, Home mechanical ventilation, Noninvasive ventilation, Disease phenotype, Mortality

## Abstract

**Purpose:**

Obesity hypoventilation syndrome (OHS) is a severe form of sleep-disordered breathing and a major contributor to chronic respiratory failure, yet optimal treatment strategies and reliable biomarkers for monitoring therapeutic efficacy remain undefined. This prospective observational study aimed to evaluate biomarker changes during the first six months of home mechanical ventilation in OHS patients and explore potential patterns.

**Methods:**

We assessed hematological, nutritional, muscle, cardiovascular, inflammatory, and fatigue-related biomarkers in OHS patients starting goal-directed noninvasive ventilation aimed to achieve normocapnia at baseline, then 1, 3, and 6 months after initiation of treatment. Predictors of five-year survival were assessed. Subgroup analyses were performed based on initiation type and apnea–hypopnea index.

**Results:**

We observed significant improvement in clinical parameters and red cell distribution width, ferritin, total protein, albumin, creatinine, creatine-kinase, total calcium, interleukin-6, and apolipoprotein A1 values during the first 6 months of treatment. Patients with low AHI and those acutely initiated exhibited persistently elevated activin A levels at six months, suggesting incomplete biological recovery and persisting inflammatory-metabolic alterations. Albumin and PaCO_2_ normalization at six months were associated with survival in exploratory analysis.

**Conclusion:**

Initiation of home mechanical ventilation is associated with versatile biomarker changes in OHS patients. Persistent differences in certain biomarkers could help identify underlying heterogeneity. Further studies are needed to validate these findings and explore the role of biomarker profiling in OHS management.

**Supplementary Information:**

The online version contains supplementary material available at https://doi.org/10.1007/s11325-026-03785-x.

## Introduction

Obesity hypoventilation syndrome (OHS) is a severe condition associated with sleep-disordered breathing and is a major contributor to chronic respiratory failure (CRF) worldwide [1]. Diagnosis is frequently made following acute hospitalizations, and affected patients experience considerable morbidity, elevated mortality and high rates of healthcare utilization [1]. Survival is greatly improved with positive airway pressure (PAP) therapy, however, it is unclear what treatment goals and markers should be monitored to optimize long-term ventilation and positively influence outcomes. Respiratory parameters, such as partial pressure of arterial CO_2_ (PaCO_2_) and bicarbonate levels are a reasonable option to monitor treatment efficacy and their normalization has been proven to improve outcomes in other long-term ventilation indications, such as COPD, however this has not yet been demonstrated in OHS [2]. Alternatively, studies have shown that apnea–hypopnea index (AHI) values may vary considerably within OHS patients and might distinguish different phenotypes, however, it is not an ideal parameter for long-term monitoring, as it reflects airway patency more than OHS disease severity.

As optimized ventilation therapy may positively influence metabolic dysfunctions inherent to the syndrome, it is possible that other, objective and quantifiable markers could also reflect treatment efficacy. Indeed, PAP therapy has been shown to alter the epigenetic regulation of multiple metabolic pathways in OHS [3] and although data remain limited, some promising biomarkers for OHS have emerged from existing studies.

Malnutrition and a reduced fat-free mass index are prevalent in CRF patients and are predictive of long-term survival [4]. In OHS patients receiving home mechanical ventilation (HMV), an altered hormonal regulation of energy balance is accompanied by improved physical function, suggesting complex interactions between nutritional status, physical function, and muscle metabolism in response to PAP therapy [5, 6]. Inflammatory pathways might pose as another potential target. Activin A, an acute-phase protein, plays a key regulatory role in numerous metabolic pathways and has been shown to predict outcomes in acute respiratory failure. Given the frequent occurrence of acute-on-chronic respiratory failure in OHS, activin A represents a promising biomarker for long-term outcome prediction in this population [7]. Other inflammatory markers have also been implicated in the pathology of CRF and the complex neurological alterations associated with fatigue [8–12]. C-reactive protein (CRP) has been identified as a predictor of outcomes in unselected HMV patients, but studies on inflammatory cytokine changes in OHS are limited, with a recent 1-month trial showing no significant changes following NIV treatment [13]. Despite this, the role of inflammation-associated and metabolic fatigue markers in OHS remains an intriguing area of investigation, particularly given the characteristic fatigue experienced by these patients. While fatigue markers have been studied in other chronic conditions, OHS has not been extensively explored in this regard. Apolipoproteins A1 and B, which are involved in lipid metabolism and reflect chronic fatigue in various diseases, may hold promise as potential targets for monitoring OHS [14]. Finally, hematological markers also demonstrate significant changes in response to PAP therapy and may serve as predictors of long-term outcomes in both CRF and OHS [15]. One such marker, red cell distribution width (RDW), is a known predictor of poor prognosis in chronic disease and has been shown to decrease following HMV treatment in CRF patients [16].

Currently, there is no data on how these potential markers reflect efficient ventilation therapy and whether they could help identify distinct phenotypes within the OHS population. This exploratory study aimed to characterize the trajectory of select biomarkers during the first 6 months of goal-directed HMV in OHS, and to explore the relationship between biomarker patterns and outcome.

## Materials and methods

### Aims and design

We conducted a prospective observational cohort study of OHS patients starting HMV with consecutive enrolment. The primary endpoint was the change in a variety of potential biomarkers at 1, 3, and 6 months after treatment initiation compared to baseline. Secondary endpoints included differences in marker changes based on initiation type (acute vs. elective), AHI level (> 30/h vs. ≤ 30/h) and therapeutic efficacy (normal vs. abnormal PaCO_2_ level after 6 months of therapy) and their association with long-term survival. Written informed consent was obtained from all participants.

### Patients

OHS patients starting HMV between January 2018 and December 2019 were evaluated. HMV was initiated either during hospitalization for acute-on-chronic respiratory failure or within an outpatient setting. Inclusion criteria were age ≥ 18 years and prior diagnosis of OHS, defined by a body mass index (BMI) ≥ 30 kg/m^2^ and daytime PaCO_2_ ≥ 45mmHgmm or relevant nighttime increase (≥ 10 mmHg) in PaCO_2_. Exclusion criteria included significant neuromuscular, pulmonary, thoracic, or metabolic comorbidities leading to hypoventilation, presence of a tracheostomy, or noncompliance with therapy. Prior chronic obstructive pulmonary disease (COPD) diagnosis was disregarded if bronchodilator free spirometry did not prove definitive diagnosis. Patients were also excluded if insufficient blood sample volume was available for biomarker analyses at any study time point. Subgroups were defined based on initiation type (acute: patients referred to the HMV program with ongoing NIV dependence after acute respiratory failure due to OHS, elective: patients referred to the HMV program with established OHS diagnosis based on anamnestic sleep study and failed CPAP titration), AHI level (> 30/h vs. ≤ 30/h) based on anamnestic sleep study value in elective cases and initial AHI recorded during NIV titration in acute cases), and therapeutic efficacy at 6 months based on PaCO2 (normal: PaCO2 < 45 mmHg; abnormal PaCO_2_ > 45 mmHg).

### Noninvasive ventilation

To ensure goal-directed long-term therapy aiming for normalized daytime PaCO_2_, we initiated patients with bilevel NIV titrated during polysomnography aided by PaCO_2_ or transcutaneous carbon dioxide (tcCO_2_) monitoring during index hospitalization according to available guidance [17, 18]. NIV was delivered using A40 ventilators (Koninklijke Philips N.V., Amsterdam) in pressure controlled, volume targeted mode via a full face or total face mask. The ventilator settings aimed for a frequency of about 2 breaths/min below the patient’s resting respiratory rate to promote a high patient-triggered breathing ratio. Expiratory positive airway pressure (EPAP) was titrated to minimize obstructive apneas (apnea–hypopnea index [AHI] < 5–10/h). The target tidal volume was initially set at 8 mL/kg, with adjustments made to achieve optimal PaCO₂/tcCO₂ levels (35–45 mmHg). If target CO₂ levels were not reached, the tidal volume was increased up to 10 mL/kg. In cases where CO₂ reduction remained inadequate, the backup rate was increased to enhance minute ventilation. We aimed for an inspiratory time/breathing cycle ratio of approximately 35%, with further adjustments based on patient comfort and synchrony. Supplemental oxygen was prescribed if partial pressure of arterial oxygen (PaO₂) was below 60 mmHg despite maintained ventilation. Treatment goals, aligned with the German HMV guidelines [19, 20], included > 80% compliance (minimum of 6 h of daily ventilator use), normalization of daytime PaCO₂ and PaO₂ levels. Follow-up visits occurred regularly (1–3 months) and involved equipment checks, verification of treatment goals, ventilator waveform analysis, and vital sign assessments. Therapy adjustments were made as needed. Patients did not receive additional interventions aimed at weight reduction or general health improvement within the study framework, and we did not control for obesity-related comorbidities or concurrent therapies.

Assessments were performed at all study time points and included anthropometric data (age, sex, BMI, initiation type, comorbidities and available diagnostic AHI values as recorded in medical charts), clinical outcome markers (pulmonary function test [PFT], arterial blood gas [ABG], Epworth sleepiness scale [ESS], Severe Respiratory Insufficiency Questionnaire summary score [SRI-SS]), ventilator data (compliance, inspiratory and expiratory positive airway pressure [IPAP and EPAP], respiratory rate, tidal volume, AHI, daily ventilation time, supplementary oxygen), functional capacity (6-min walking test [6MWT]) and laboratory samples (venous blood sampling for biomarker assessment [21, 22]). PFTs were performed with the Piston PinkFlow meter (Piston Ltd, Budapest, Hungary) according to the American Thoracic Society and European Respiratory Society guidelines [23]. ABG samples were obtained at least 15 min after discontinuing ventilation and/or oxygen supplementation. Laboratory parameters were determined from standard venous blood draw. Blood samples were obtained during routine clinical visits (morning sampling for inpatients and fasting status for outpatients) and stored and processed according to standard laboratory protocols. Long-term survival was assessed 5 years after initial study enrolment and was based on electronic medical records.

### Studied biomarkers

To best reflect the key pathophysiological domains affected in OHS, we selected markers that were both easily identifiable and relatively stable. These included indicators of nutritional status (total protein, albumin, triglyceride, cholesterol, transferrin), muscle metabolism (creatinine, blood urea nitrogen [BUN], creatin-kinase [CK], total serum calcium, phosphate), inflammation and potential fatigue-related markers (white blood cell [WBC], CRP, interleukin-6 [IL-6], interleukin-1 receptor antagonist [IL-1ra], soluble tumor necrosis factor receptor II [sTNF RII], activin A, apolipoprotein A1, apolipoprotein B, lipoprotein), hematological parameters (red blood cell count [RBC], RDW, hemoglobin, hematocrit, platelet count [PLT], ferritin, total iron-binding capacity [TIBC]), and cardiovascular stress (N-terminal pro-brain natriuretic peptide [NT-proBNP], high sensitivity troponin I [HS troponin I], high-density lipoprotein [HDL], low-density lipoprotein [LDL]), based on prior studies in acute and chronic respiratory failure.

Routine laboratory parameters were measured during automated laboratory analysis (Sysmex XN-1000 Hematological Analyser [Sysmex, Kobe, Japan], AU 5801 Clinical Chemistry Analyser [Beckman Coulter, California, USA], Cobas e 801 Analytical Unit [Roche Diagnostics, Basel, Switzerland]). Serum samples leftover from routine laboratory assessment were frozen and stored at −20 °C. The additional measurements were performed by commercial enzyme-linked immunoassay (ELISA) kits (Human IL-6 Quantikine HS ELISA Kit, HS600C; Human IL-1ra/IL-1F3 Quantikine ELISA Kit, DRA00B; Human TNF RII/TNFRSF1B Quantikine ELISA Kit, DRT200; Human/Mouse/Rat Activin A Quantikine ELISA Kit, DAC00B; R&D Systems, Minneapolis, Minnesota, US). ELISA measurements of all study samples were performed at the same time and according to the manufacturer instructions.

### Statistical analysis

Continuous data are presented as median (interquartile range), and categorical data as n (percentages). The normality of data was assessed using the Shapiro-Wilks test. Based on these results, we used nonparametric tests for the evaluation (the Mann–Whitney test for the comparison of continuous variables, and the Chi-square test for categorical variables). For comparisons between baseline and follow-up results, we used the Wilcoxon signed-rank test or Friedman ANOVA by ranks. Spearman correlation was performed between changes in biomarker levels and classic clinical markers. The Kaplan–Meier graph was generated to assess 5-year survival, and the univariate comparison of the probability of death according to different groupings was performed with the log-rank test. Missing values were not included in the analysis. Significance level was set at *p* < 0.05.

## Results

**Fig. 1:**
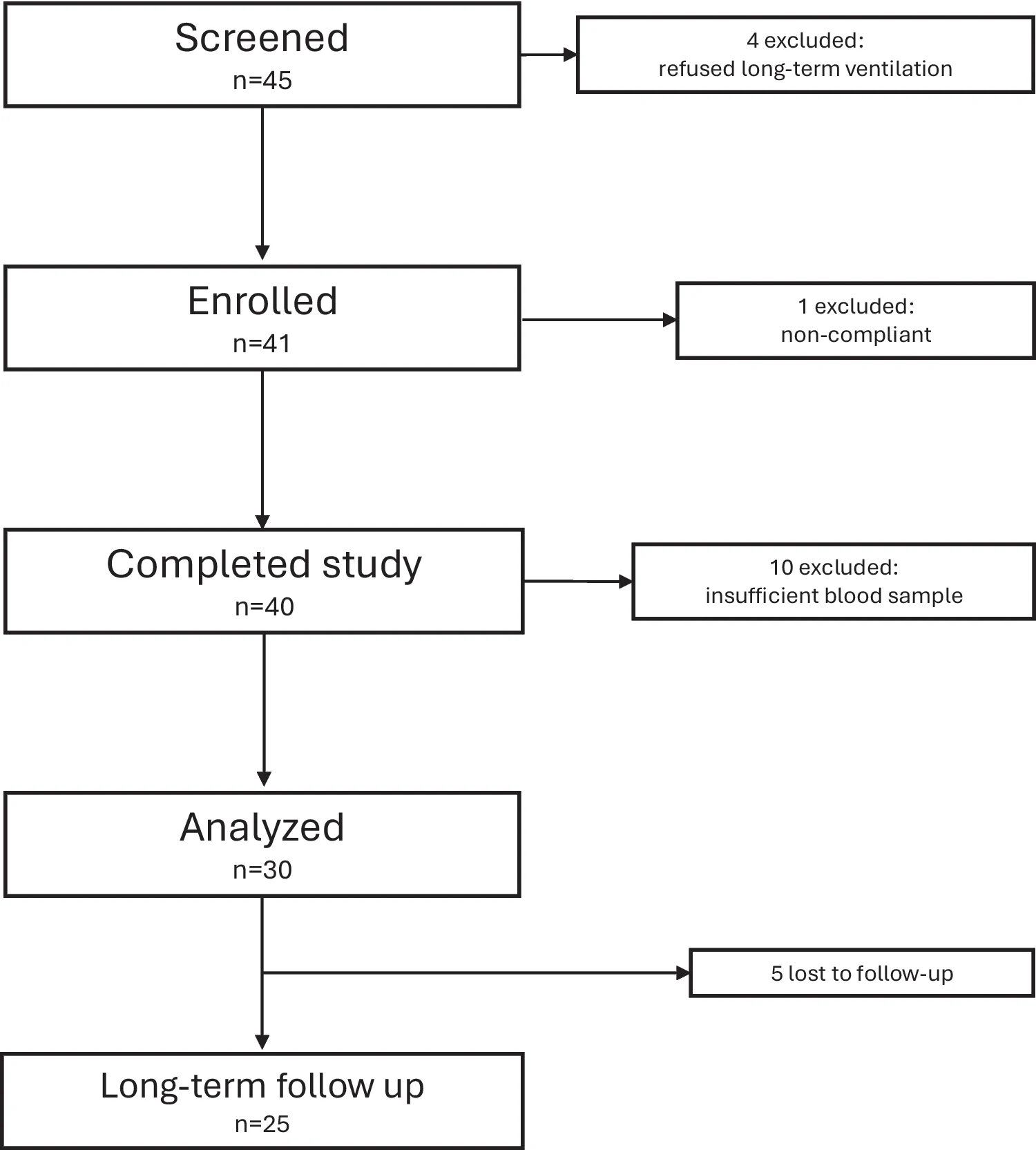
Flowchart of study patients

Of the 45 patients screened, 40 completed the initial study and 30 had sufficient samples from all time points to be included in the analysis (see Fig. 1). 5-year follow-up data was available for 25 patients.

Baseline characteristics of the study population are presented in Table 1. Patients were predominantly male, severely obese, and had multiple comorbidities. COPD misdiagnosis was common, with 6 (20%) patients showing no significant obstruction on bronchodilator free spirometry, despite prior medical history listing the condition based on hypercapnia. Half of the cohort was recruited following an episode of acute-on-chronic respiratory failure, while the remaining 50% were enrolled during an elective admission for initiation of HMV. Acutely and electively enrolled patients showed no differences in anthropometric data or most clinical parameters, however, acutely enrolled patients required more supplementary oxygen and had lower AHI and higher PaCO_2_ values compared to their electively enrolled counterparts (see Supplementary Table 1). On the other hand, we found no significant differences when comparing patients with AHI > 30/h (*n* = 12 (40%)) or AHI ≤ 30/h (*n* = 18 (60%)), apart from their AHI values (see Supplementary Table 2).

**Table 1:** Baseline parameters of the study population

	All patients (*n* = 30)
Anthropometric data	
Age (years)	55.0 (14.0)
Male sex	22 (73.3%)
BMI (kg/m^2^)	43.3 (14.3)
Comorbidities	
HTN	25 (83.3%)
CHF	7 (76.7%)
PHT	4 (13.3%)
CAD	7 (23.3%)
DM	14 (46.7%)
Smoking status	9 (30.0%)
Smoking intensity (pack years)	40 (36)
Ventilator settings	
IPAP (cmH_2_O)	22.4 (6.4)
EPAP (cmH_2_O)	11.7 (4.0)
Respiratory rate (1/min)	16.0 (2.4)
Tidal volume (mL)	685 (170)
Supplementary O_2_ (L/min)	2.0 (3.3)
Daily ventilation (hour)	7.5 (2.4)
Clinical parameters	
AHI (/h)	19.4 (49.4)
pH	7.41 (0.04)
PaO_2_ (mmHg)	60.7 (15.9)
PaCO_2_ (mmHg)	48.7 (13.9)
HCO_3_ mmol/L	30.4 (7.4)
FVC%	65.5 (33.0)
FEV1%	60.5 (36.0)
FEV1/FVC%	93.0 (18.0)
PEF%	54.5 (31)
6MWT (m)	252 (174)
ESS (points)	10 (9)
SRI-SS	57.9 (27.0)

Baseline demographics for all study patients. Abbreviations: *AHI* apnea–hypopnea index, *BMI* body mass index, *COPD* chronic obstructive pulmonary disease, *HTN* hypertension, *CHF* chronic heart failure, *CAD* coronary artery disease, *DM* diabetes mellitus, *PHT* pulmonary hypertension, *IPAP* inspiratory positive airway pressure, *EPAP* expiratory positive airway pressure, *AHI* apnea hypopnea index, *PaO*_*2*_ arterial oxygen pressure, *PaCO*_*2*_ arterial carbon dioxide pressure, *HCO*_*3*_ bicarbonate, *FVC%* percentage of expected forced vital capacity, *FEV1%* percentage of expected 1 s forced expiratory volume, *FEV1/FVC%* percentage of expected 1 s forced expiratory volume/forced vital capacity, *PEF%* percentage of expected peak expiratory flow, *ESS* Epworth sleepiness scale, *SRI-SS* Severe Respiratory Insufficiency Questionnaire summary score, *6MWT* 6 min walking test

### Changes in clinical parameters during the study

**Fig. 2:**
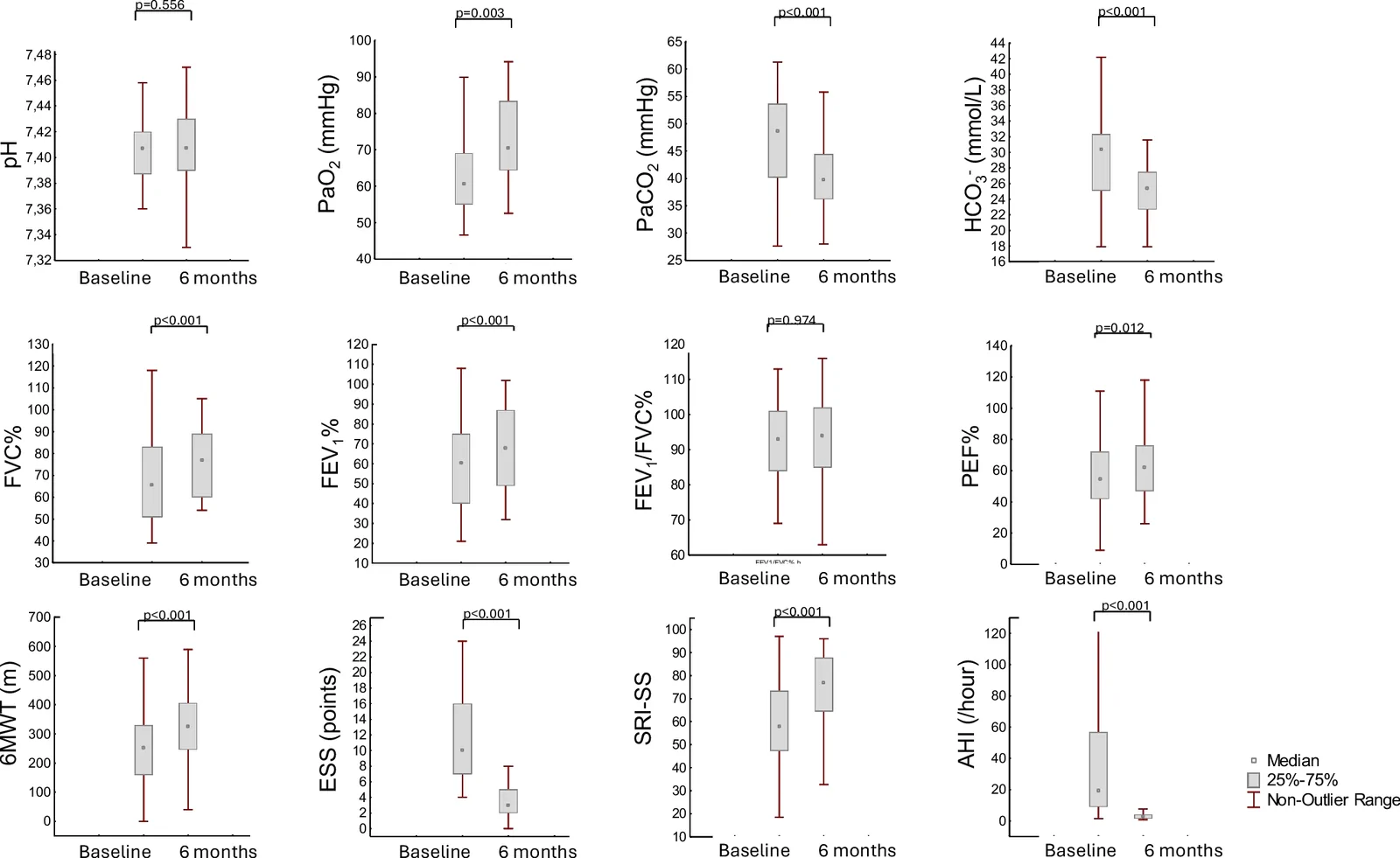
Routine clinical marker changes during the study period. Boxplot graph of clinical parameters at baseline and 6 months of home mechanical ventilation treatment. Abbreviations: AHI: apnea hypopnea index, PaO_2_ arterial oxygen pressure, PaCO_2_: arterial carbon dioxide pressure, HCO_3_^−^: bicarbonate, FVC%: percentage of expected forced vital capacity, FEV1%: percentage of expected 1 s forced expiratory volume, FEV1/FVC%: percentage of expected 1 s forced expiratory volume/forced vital capacity, PEF%: percentage of expected peak expiratory flow, ESS: Epworth sleepiness scale, SRI-SS: Severe Respiratory Insufficiency Questionnaire summary score, 6MWT: 6 min walking test

During the six-month follow-up, patients demonstrated significant clinical improvement. BMI decreased from 44.4 (9.7) to 41.0 (8.5) kg/m^2^ (*p* = 0.044). Daily ventilator use decreased (8.2 (2.4) to 6.6 (1.2) hours, *p* < 0.001), while ventilator settings remained largely unchanged. O_2_ supplementation also decreased from 2.4 (2.5) to 1.1 (1.3) L/min, *p* = 0.005. Most clinical parameters improved significantly over the study period, with the exception of FEV1/FVC% and pH (see Fig. 2 and Supplementary Table 2 for detailed values). The therapeutic goal (e.g. PaCO_2_ < 45 mmHg) was achieved in 25 patients (83,3%) at the 6-month mark.

Subgroup analyses showed that initiation type was associated with different baseline values and that improvements occurred in both acute and elective groups, although changes were generally more pronounced among acutely initiated patients (see Supplementary Table 2). After six months of HMV, clinical markers were largely comparable between groups, with the exception of pH and ESS values. Acutely initiated patients had lower pH (7.40 (0.04) vs. 7.42 (0.04), *p* = 0.002) and ESS scores (2.0 (3.0) vs. 4.0 (5.0), *p* = 0.016) compared to elective patients at 6 months. Acute patients were also less likely to achieve normocapnia by the 6-month mark compared to elective patients (66,6 vs. 100%, *p* = 0.014), despite optimized therapy.

In contrast, low and high AHI grouping was not associated with apparent differences in baseline or 6-month clinical parameters (detailed analysis not shown) other than baseline AHI values (9.1 (11.1) vs. 71.1 (58.1)/h, *p* < 0.001).

### Biomarker trends over time

Among the biomarkers measured longitudinally, several demonstrated significant changes across the entire cohort (Table 2). These included RDW, ferritin, total protein, albumin, creatinine, CK, total calcium, IL-6, and apolipoprotein A1.

**Table 2:** Biomarker changes for the whole study population

Marker	Baseline	1 month	3 months	6 months	*p* value
Hematological					
RBC (T/L)	5.2 (1.0)	5.3 (0.9)	4.8 (1.0)	4.8 (0.9)	***p* = 0.027**
RDW (%)	15.1 (3.3)	14.7 (2.7)	14.7 (2.0)	13.5 (1.3)	***p* < 0.001**
Hb (g/L)	147.5 (33.0)	153.0 (26.0)	144.0 (27.0)	143.5 (23.0)	*p* = 0.093
Hct (L/L)	0.46 (0.10)	0.46 (0.07)	0.43 (0.07)	0.42 (0.06)	***p* = 0.012**
PLT (G/L)	246.0 (129.0)	230.0 (115.3)	243.5 (128.0)	244.0 (88.0)	*p* = 0.808
Ferritin (umol/L)	13.3 (7.2)	16.9 (11.7)	13.6 (12.6)	11.5 (5.7)	***p* = 0.007**
TIBC (umol/L)	70.7 (29.7)	73.5 (14.3)	71.0 (21.2)	74.7 (17.0)	*p* = 0.101
Nutrition					
Total protein (g/L)	67.4 (7.5)	73.2 (4.5)	73.8 (5.2)	73.8 (7.3)	***p* < 0.001**
Albumin (g/L)	38.7 (7.1)	43.0 (2.6)	42.5 (3.9)	43.9 (3.8)	***p* < 0.001**
TG (mmol/L)	2.0 (1.1)	2.2 (1.5)	2.0 (1.2)	2.2 (1.8)	*p* = 0.277
Cholesterol (mmol/L)	4.9 (1.4)	5.0 (1.8)	4.8 (1.2)	5.0 (1.1)	*p* = 0.192
Transferrin (g/L)	2.90 (1.06)	2.96 (0.67)	3.0 (0.7)	3.0 (0.7)	*p* = 0.117
Na (mmol/L)	140.0 (5.0)	140.0 (3.0)	140.0 (3.3)	140.0 (4.0)	*p* = 0.547
K (mmol/L)	4.4 (0.7)	4.3 (0.6)	4.3 (0.5)	4.5 (0.5)	*p* = 0.056
Mg (mmol/L)	0.81 (0.51)	0.80 (0.13)	0.79 (0.11)	0.83 (0.13)	*p* = 0.883
Muscle					
Creatinine (umol/L)	69.5 (21.0)	74.5 (31.0)	78.5 (4.1)	84.0 (39.0)	***p* = 0.001**
BUN (mmol/L)	6.2 (3.4)	6.5 (3.4)	7.0 (4.1)	7.3 (3.6)	*p* = 0.239
CK (U/L)	76.0 (58.0)	110.0 (87.3)	115.5 (92.0)	142.5 (87.0)	***p* < 0.001**
Total calcium (mmol/L)	2.4 (0.2)	2.5 (0.2)	2.46 (0.09)	2.5 (0.1)	***p* < 0.001**
Phosphate (mmol/L)	1.11 (0.29)	1.13 (0.25)	1.12 (0.19)	1.11 (0.20)	*p* = 0.966
Cardiovascular					
NT-proBNP (pg/mL)	177.3 (483.6)	92.2 (345.1)	87.9 (263.1)	74.5 (195.8)	*p* = 0.398
HS troponin I (ng/L)	5.1 (24.8)	4.1 (10.7)	7.1 (22.7)	4.8 (7.7)	*p* = 0.113
CKMB (U/L)	12.0 (9.0)	11.0 (10.0)	12.0 (8.3)	13.0 (4.0)	*p* = 0.538
HDL (mmol/L)	1.0 (0.4)	1.1 (0.5)	1.1 (0.4)	1.2 (0.3)	*p* = 0.257
LDL (mmol/L)	3.1 (1.1)	3.1 (1.2)	3.0 (0.6)	3.1 (0.9)	*p* = 0.625
Lipoprotein (g/L)	0.11 (0.51)	0.11 (0.44)	0.12 (0.39)	0.12 (0.44)	*p* = 0.614
Inflammation and fatigue					
WBC (G/L)	7.9 (2.9)	8.5 (2.6)	8.7 (2.6)	8.3 (2.7)	***p* = 0.028**
CRP (mg/L)	10.5 (21.7)	9.9 (9.5)	8.3 (11.4)	8.4 (7.9)	*p* = 0.136
IL-6 (pg/mL)	5.8 (10.3)	3.9 (5.8)	2.9 (6.4)	2.5 (4.9)	***p* = 0.048**
IL-1ra (pg/mL)	1151.4 (1921.7)	907.7 (1826.1)	813.5 (815.7)	1052.2 (1067.2)	*p* = 0.515
sTNF RII (pg/mL)	3756.6 (1788.4)	3753.5 (1372.0)	3767.7 (1889.3)	3755.1 (1668.0)	*p* = 0.871
Activin A (pg/mL)	340.9 (118.9)	366.2 (133.9)	400.7 (231.7)	392.4 (227.4)	*p* = 0.555
ApoA1 (g/L)	1.36 (0.38)	1.37 (0.35)	1.31 (0.46)	1.36 (0.35)	***p* = 0.035**
ApoB (g/L)	0.87 (0.35)	0.92 (0.34)	0.88 (0.26)	0.87 (0.35)	*p* = 0.355

Biomarker changes during the study period for all patients with Friedman ANOVA *p* values listed. Significant data are marked with bold font. Abbreviations: *ApoA1* apolipoprotein A1, *ApoB* apolipoprotein B, *BUN* blood urea nitrogen, *CRP* C-reactive protein, *CK* creatine kinase, *CKMB* creatine kinase myoglobin-binding, *Hb* hemoglobin, *Hct* hematocrit, *HDL* high-density lipoprotein, *HS troponin I* high sensitivity troponin I, *IL-6* interleukin-6, *IL-1ra* interleukin-1 receptor antagonist, *LDL* low-density lipoprotein, *NT-proBNP* N-terminal pro-brain natriuretic peptide, *RBC* red blood cell, *RDW* red cell distribution width, *sTNF RII* soluble tumor necrosis factor receptor II, *TG* triglyceride, *TIBC* total iron binding capacity, *WBC* white blood cell

Subgroup analysis revealed that initiation type and AHI grouping corresponded to distinctly different baseline biomarker profiles (baseline parameters are summarized in Supplementary Table 3). Acutely enrolled patients had lower hemoglobin, total protein, albumin, cholesterol, HDL, total calcium, and apolipoprotein A1 and also exhibited higher levels of IL-6, IL-1ra, sTNF RII, and activin A values compared to electively enrolled patients.

High AHI patients had higher Hb, ferritin, protein, albumin, CK, calcium and lower RDW and sTNF RII values compared to low AHI patients (see Supplementary Table 3). Other inflammation and fatigue markers also showed a tendency to be lower in high AHI patients, with Il-6, IL-1ra and activin A showing near-significant differences.

**Fig. 3:**
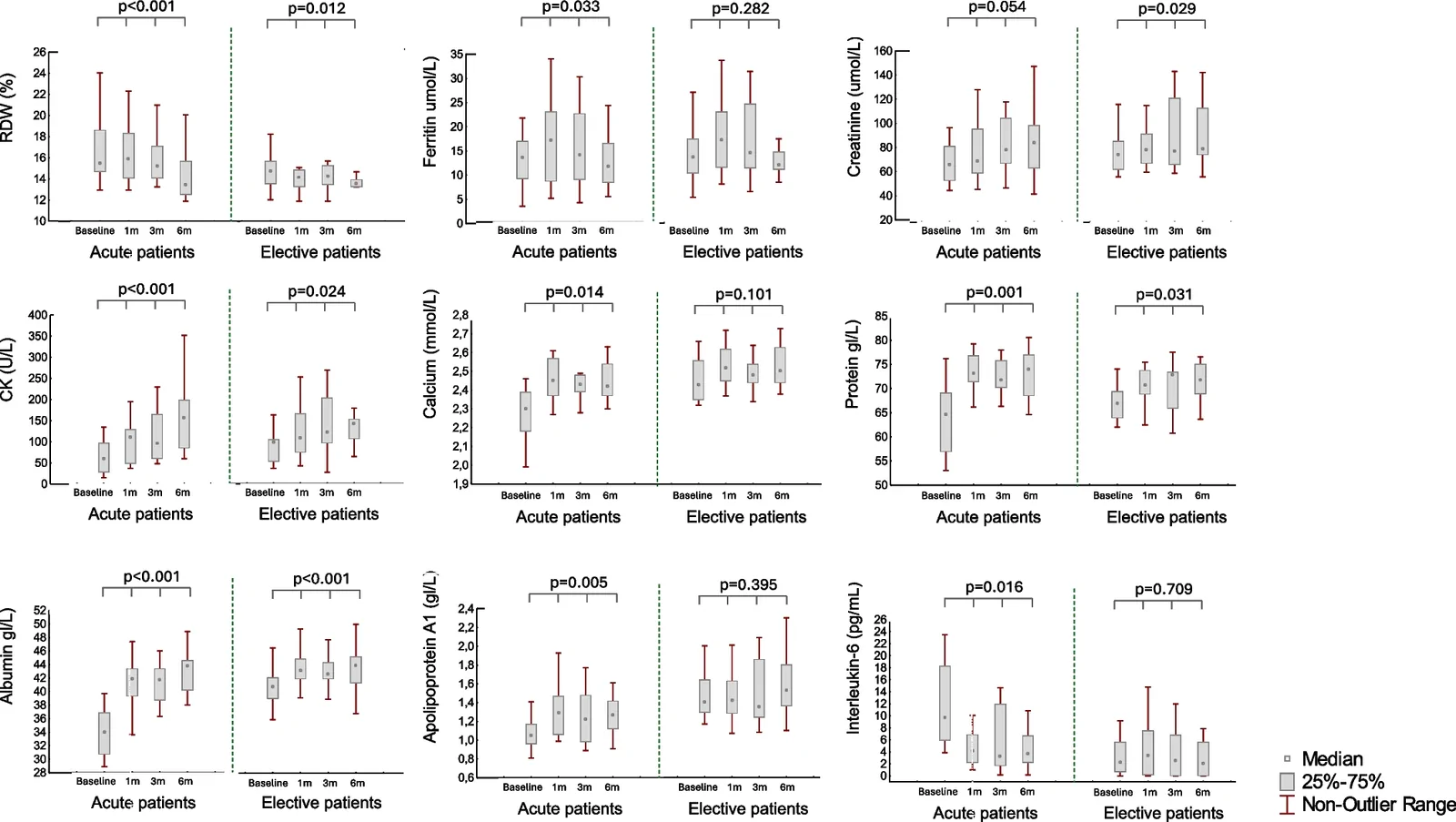
Subgroup analysis (acute and elective patients) of biomarkers showing significant change during the first 6 months of home mechanical ventilation. Boxplot graph of biomarkers at baseline, 1 month, 3 months, and 6 months of home mechanical ventilation treatment for acutely initiated and electively initiated patients. Friedman ANOVA by Ranks p-values are marked. Abbreviations: RDW: red cell distribution width, CK: creatine-kinase

Further subgroup analysis revealed that RDW, CK, and total protein levels changed significantly in both acute and elective groups (see Fig. 3). Creatinine levels showed a near-significant increase in acute patients (*p* = 0.054) and a significant increase in elective patients (*p* = 0.029). Changes in ferritin, albumin, calcium, apolipoprotein A1, and IL-6 were significant only in the acutely initiated group, but not the elective patients.

Most biomarkers that initially differed by initiation type or AHI group no longer showed significant differences after six months of HMV, with some exceptions. Apolipoprotein A1 and activin A remained significantly different between initiation groups at the six-month mark (ApoA1: 1.27 ± 0.19 vs. 1.58 ± 0.35, *p* = 0.006; activin A: 461.3 ± 112.1 vs. 323.3 ± 150.5, *p* = 0.011 for acute vs. elective, respectively). Notably, CRP levels, which were similar at baseline, differed significantly at six months between initiation groups (acute: 12.9 ± 10.3 vs. elective: 6.5 ± 3.3, *p* = 0.034). There were also significant differences persisting at the 6-month mark between AHI groups (high vs. low) regarding CKMB (15.0 (4.0) vs. 11.5 (5.0), *p* = 0.048), sTNF RII (3300.8 (1175.3) vs. 3807.1 (1433.9), *p* = 0.048), and activin A (332.7 (218.9) vs. 463.2 (414.7), *p* = 0.022) levels.

### Correlation of clinical parameters and biomarkers, and association with clinical outcomes

Most changes of relevant biomarkers showed significant correlations with some clinical parameter changes (see Table 3). AHI change showed the most correlations with biomarker changes.

**Table 3:**
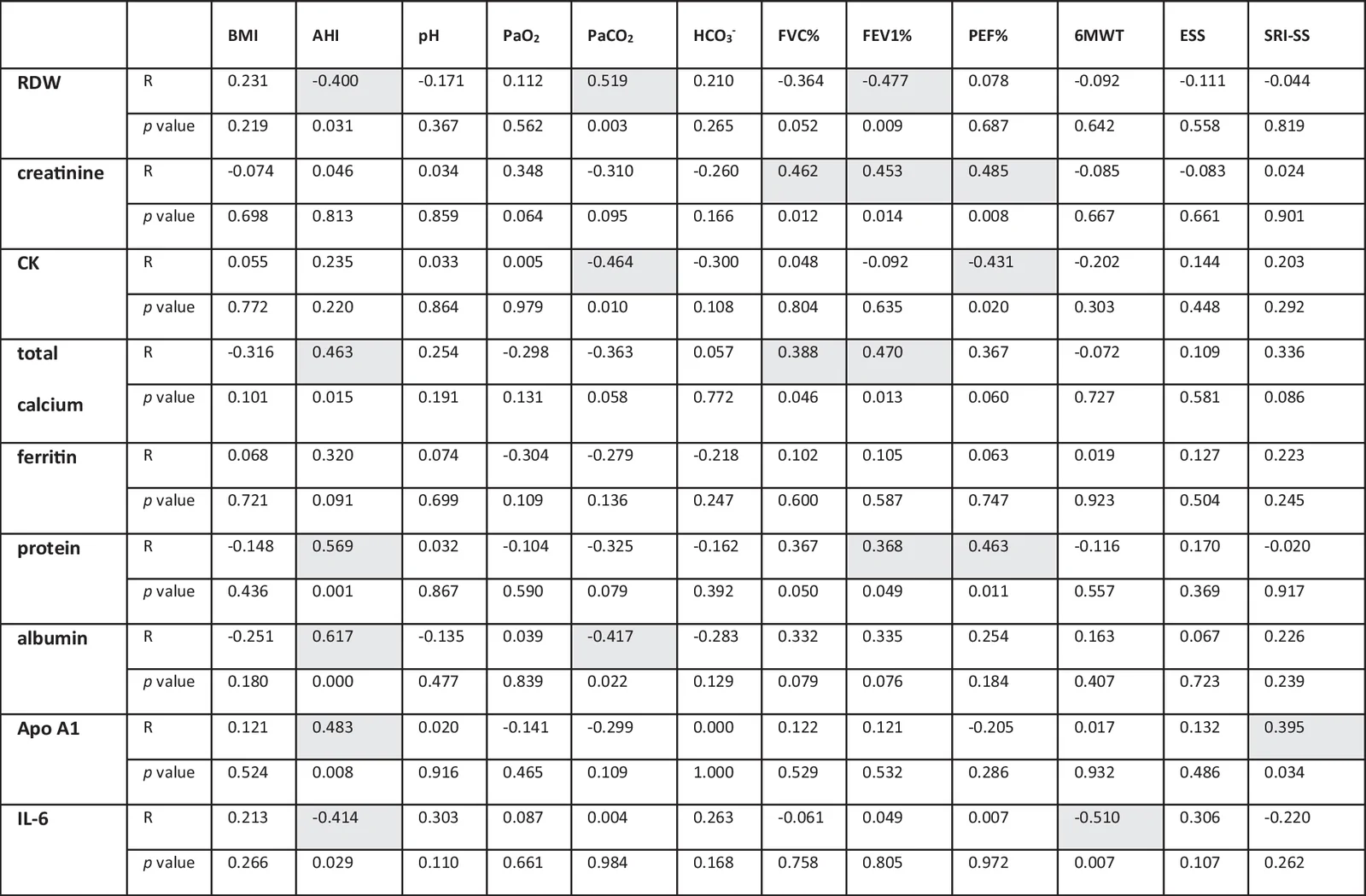
Correlation between changes in relevant biomarkers and key clinical outcomes throughout the study period

Spearman correlation rho values for changes in relevant biomarkers and classic outcome markers. Shading indicates significant association (*p* < 0.05). Abbreviations: *ApoA1* apolipoprotein A1, *CK* creatine kinase, *IL-6* interleukin-6, *RDW* red cell distribution width, *AHI* apnea hypopnea index, *PaO*_*2*_ arterial oxygen pressure, *PaCO*_*2*_ arterial carbon dioxide pressure, *HCO*_*3*_^*−*^ bicarbonate, *FVC%* percentage of expected forced vital capacity, *FEV1%* percentage of expected 1 s forced expiratory volume, *FEV1/FVC%* percentage of expected 1 s forced expiratory volume/forced vital capacity, *PEF%* percentage of expected peak expiratory flow, *ESS* Epworth sleepiness scale, *SRI-SS* Severe Respiratory Insufficiency Questionnaire summary score, *6MWT* 6 min walking test

When assessing 5-year survival, 5 patients were lost to follow-up because they discontinued therapy (therapy length was 22.5 ± 7.2 months). Out of the 25 patients available for long-term survival analysis, 3 died during the assessment period (survival was 32.3 ± 15.3 months). All non-survivors were from the acute initiation group, one out of the three deaths was COVID related, but this occurred outside of the study period. When comparing non-survivors and survivors, the only significant difference in baseline clinical parameters was PEF (31 (22) vs. 56 (25) *p* = 0.002), however the difference in baseline PaCO_2_ (54.8 (40.7) vs 47.5 (14.5) *p* = 0.072) and HCO_3_^−^ (32.7 (11.7) vs 29.7 (7.6) *p* = 0.072) was near-significant. Survivors also differed from non-survivors in the aspect that they were more likely to be normocapnic at 6 months (88.9% vs 33.3% *p* = 0.014). There was also a tendency for survivors to be electively initiated compared to non-survivors (55.6% vs 0% *p* = 0.068).

**Fig. 4:**
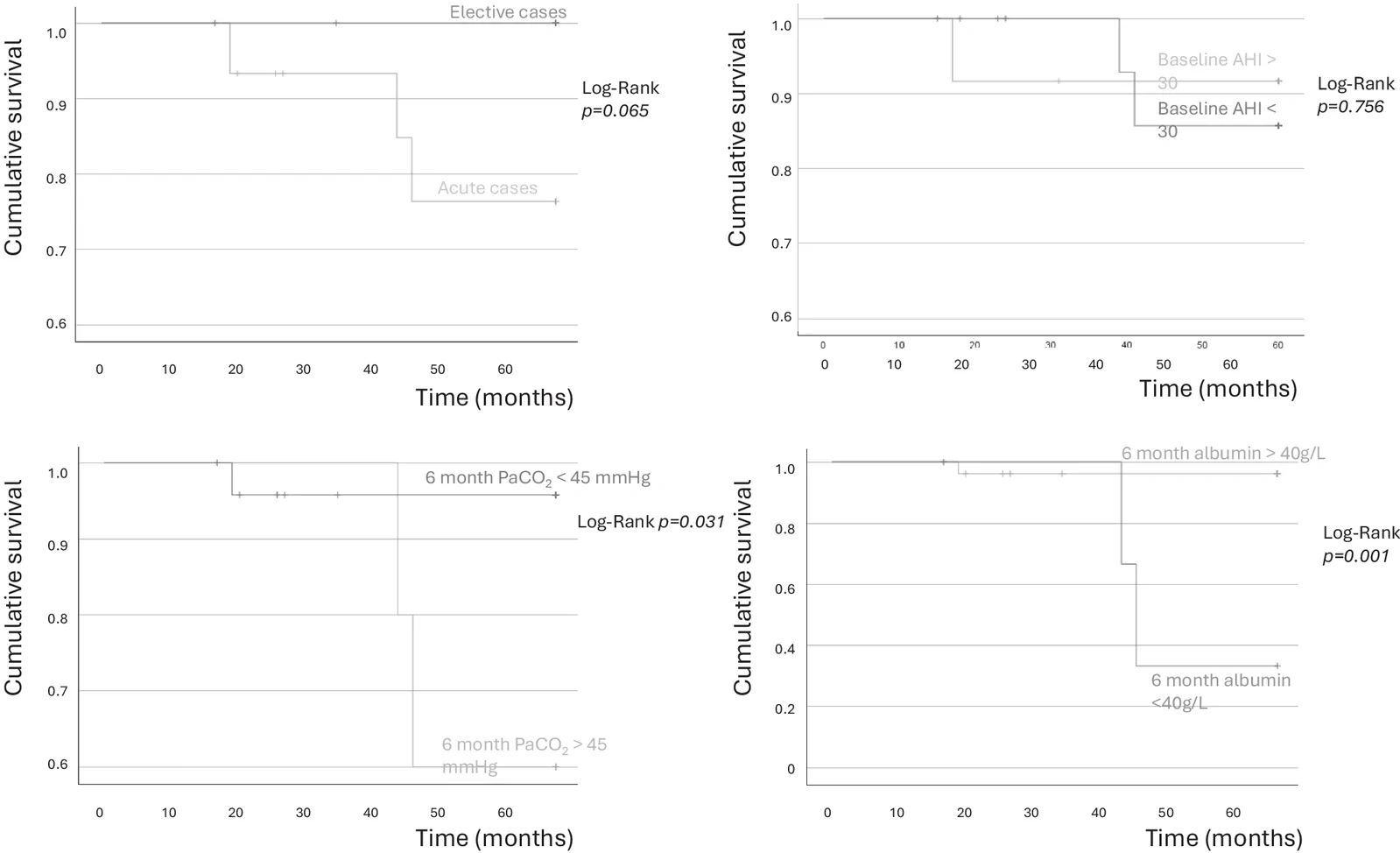
Survival of study patient by subgroups. Abbreviations: AHI: apnea hypopnea index, RDW: red cell distribution width, PaCO_2_: arterial carbon dioxide pressure

The Kaplan Meier curves for different groups based on initiation type, baseline AHI level, 6-month albumin and 6-month PaCO_2_ levels are depicted in Fig. 4. The log-rank p-value was 0.031 for the comparison of patients with normocapnia and hypercapnia at the 6-month mark, and 0.001 for the comparison of patients with albumin below or above 40 g/L at the 6-month mark, indicating a significant difference between the survival distributions. Median survival times could not be calculated because of the low mortality rate.

## Discussion

In this study, we aimed to explore key biomarker profile changes in patients with OHS during six months of goal-directed noninvasive ventilation treatment. We found that goal-directed HMV was not only associated with significant improvements in clinical parameters, but also hematological, nutritional, inflammation and muscle metabolism biomarkers. Additionally, we found that biomarker profiles showed persistent differences in patients starting ventilation electively or after an episode of acute-on-chronic respiratory failure, suggesting potential biological heterogeneity within OHS.

The optimal approach to treating OHS remains a subject of debate. Current guidelines recommend a CPAP trial in patients with an AHI > 30, though some evidence indicates that NIV offers greater benefits, including more pronounced improvements in PaCO₂, FEV₁, and 6MWT, as well as reductions in pulmonary hypertension[24–26]. NIV is typically reserved for patients with low AHI, those already on ventilatory support following acute exacerbations, or patients with persistent hypoxemia despite CPAP [27]. Presumably, other patients might also benefit from NIV, but additional markers may be needed to guide individualized treatment and improve long-term outcome.

In line with the aforementioned evidence, we observed significant improvement in gas exchange, exercise capacity and quality of life in our cohort of OHS patients receiving goal-directed NIV aimed to achieve normocapnia. As an important observation, we found that clinical parameters were largely comparable after 6 months of NIV in all patient groups, suggesting that aiming for normocapnia in OHS yields favorable clinical outcome regardless of disease severity. This is in line with a recent large database analysis that found that normocapnia improved long term outcome in OHS [28].

Chronic respiratory failure has been shown to induce nutritional abnormalities, loss of muscle mass, systemic inflammation and protein synthesis/breakdown disbalance [29]. Long-term ventilation may effectively reduce work of breathing and positively influence these metabolic pathways. Parallel to clinical parameter improvements, we observed significant changes in RDW, ferritin, total protein, albumin, creatinine, CK, total calcium, IL-6, and apolipoprotein A1 values, suggesting that HMV not only stabilizes respiratory function, but may also contribute to systemic recovery. The observed biomarker trends provide additional insight into the physiological changes associated with HMV. The significant reduction in inflammatory markers such as IL-6 and CRP, particularly in acutely initiated patients, suggests a dampening of systemic inflammation. Improvements in nutritional markers (e.g., albumin, total protein) and muscle metabolism indicators (e.g., creatinine, CK) further support the hypothesis that HMV facilitates metabolic and functional recovery.

Budweiser et al. reported diminishing hemoglobin values in OHS patients receiving NIV treatment. Importantly, the follow-up visit in that study was not at a standardized time point and the study population only included an estimated 18% of acutely initiated patients [15]. In our study with a more balanced patient distribution, we found that hemoglobin differed significantly based on initiation type and that hemoglobin change was non-linear during the whole of the study period. The dynamic of hemoglobin change might suggest a transient increase in erythropoiesis (also reflected in ferritin and TIBC values) after initiation of HMV. The changes overall result in a robust RDW reduction in both acute and elective groups, in line with previously published data showing similar change in an unselected CHF population starting NIV [16]. Additional mechanisms of RDW reduction during long-term ventilation are also possible, as this marker is influenced by several pathways, such as oxidative stress, inflammation and nutritional status [30]. Given that RDW is a reliable mortality predictor in several patient groups, it may serve as a prognostic marker in OHS as well [30, 31]. We did not find RDW change to be predictive of survival in our cohort, however, this might have been the result of excellent control of blood gas values and low overall mortality. A previous study has shown no RDW change with only CPAP therapy in a cohort of OSAS patients, where baseline BMI values were in the range of OHS [32]. Future studies are needed to determine whether RDW reduction during HMV treatment may help optimize treatment and predict outcome.

We found increasing total protein, albumin, and CK values in both acutely and electively initiated OHS patients after 6 months of HMV. This suggests improving nutritional status and muscle metabolism, findings that aligned with improvements in physical function. Albumin, in particular, correlated with mortality in our cohort, consistent with its established role as a negative acute-phase protein, as well as inflammation, nutrition and general prognostic marker [33]. Interestingly, albumin change showed the highest correlation to PaCO_2_ change in our study. This again highlights the possibility that goal-directed therapy in OHS might optimize long term outcomes similar to other chronic respiratory conditions such as COPD [2].

In addition to albumin, several of the longitudinal marker changes in our cohort may reflect a complex interplay of pathophysiological pathways, such as reduced hypoxemia and hypercapnia, metabolic recovery, as well as reduced inflammation. Ferritin, in addition to reflecting iron stores and erythropoiesis activity, is also an acute-phase protein and inflammation marker. It has been shown to be elevated in obesity, an effect likely more indicative of inflammation rather than iron deficiency [34]. Apolipoprotein A1, a major component of HDL, exerts anti-inflammatory and antioxidant effects and has been linked to cardiometabolic risk and chronic inflammatory states [35]. Its increase may indicate restoration of lipid metabolism and attenuation of inflammatory processes. The presence of systemic inflammation in OHS has already been established, as reflected by elevated levels of IL-6 compared to obese patients without hypoventilation [36, 37]. We found a significant reduction in IL-6 and a concurrent increase in apolipoprotein A1 over six months, primarily driven by improvements in acutely initiated patients. This is in contrast with findings from Borel et al., who observed no change in IL-6 over one month of therapy in ambulatory patients with milder OHS. Our results suggest that the degree of inflammation and response to treatment may differ according to disease severity and initiation context [13]. Despite previous evidence that numerous inflammatory markers play a role in fatigue in several conditions, inflammatory marker changes in our study did not correlate with changes in subjective measures of sleepiness (ESS) but instead showed stronger correlations with objective functional measure (6MWT) and health related quality of life (SRI-SS) change. This is consistent with earlier findings suggesting that inflammatory activity in OHS is more closely related to physical performance than perceived fatigue [8].

Despite overall convergence in clinical parameters and most biomarkers between acutely and electively initiated patients, persistent disparities in apolipoprotein A1 and activin A levels may reflect underlying differences in metabolic or inflammatory status that are not fully resolved by HMV. Apolipoprotein A1, a protective anti-inflammatory and antioxidant molecule, remained significantly lower in acutely initiated patients, potentially reflecting unresolved metabolic stress or systemic inflammation [35]. Similarly, elevated activin A, a marker involved in muscle wasting and metabolic regulation that is associated with poor prognosis in acute respiratory failure, remained elevated in the same group, suggesting incomplete biological recovery despite clinical stabilization [7]. Importantly, low baseline AHI was also associated with persistently higher activin A levels, despite apparent clinical improvement. Given that low-AHI OHS patients are more likely to present with acute respiratory failure and may respond better to NIV than CPAP, these biomarker differences may reflect a distinct pathophysiological profile [27].

Together, these findings suggest that biomarker assessment may be warranted in OHS management. RDW and albumin may serve as early indicators of treatment response, while inflammatory markers may identify residual disease activity, heightened systemic inflammation and metabolic dysregulation. These biomarker patterns may be important targets for future research focusing on more personalized treatment strategies.

Although this is the most comprehensive biomarker study in OHS to date, given the relatively small sample size and the number of biomarkers assessed without formal correction for multiple comparison, the risk of type I error must be considered, and findings should be interpreted as hypothesis-generating. While these results are promising, several further limitations must be acknowledged. First, we did not control for weight-related comorbidities and therapies, which might influence the overall results. These considerations are especially relevant given the growing use and complex metabolic effect of GLP-1 agonists, although the study period predated their generalized use. Second, due to the heterogeneity of patient presentation, AHI values were not derived from standardized baseline PSG within the study but from prior sleep studies (elective) or during NIV titration (acute), limiting comparability between groups. Third, the study coincided with the COVID-19 pandemic and although only one death was COVID-related, the pandemic may have also influenced healthcare access, follow-up, and adherence. A larger sample size and more homogenous sampling would permit the use of further multivariate or more advanced statistical approaches to identify biomarker clusters and possible confounders. Future studies should validate our findings in larger cohorts and assess the predictive utility of these biomarkers and their role in tailoring therapy.

In conclusion, initiation of goal-directed home mechanical ventilation in OHS patients results in significant changes across hematologic, nutritional, muscular, cardiovascular and inflammatory biomarker domains. Notably, activin A levels continue to distinguish acutely and electively initiated patients after six months of treatment, suggesting persistent biological differences. Future studies should assess the prognostic value of these markers and explore their use as therapeutic targets in the management of OHS.

## Supplementary Information

Below is the link to the electronic supplementary material.

Supplementary file1 (DOCX 46 KB)

## Data Availability

The datasets analyzed during the current study are available from the corresponding author on reasonable request.
