# Supplementary material for: Biomarker changes during long-term noninvasive ventilation in obesity hypoventilation syndrome: a prospective observational study
Source: Sleep Breath. 2026 Aug 24;30(5):241. doi: 10.1007/s11325-026-03785-x (PMC13503410; doi:10.1007/s11325-026-03785-x)
Supplement: Supplementary file 1 [file 11325_2026_3785_MOESM1_ESM.docx]

Title: Biomarker changes during long-term noninvasive ventilation in obesity hypoventilation syndrome: a prospective observational study

Authors: Luca Valko, Vivien Moro, Szabolcs Baglyas, Blanka Mezo, Eszter Podmaniczky, Zoltan Prohaszka, Andras Lorx

Journal: Sleep and Breathing

Corresponding author: Luca Valko ([valko.luca@semmelweis.hu](mailto:valko.luca@semmelweis.hu)), Department of Anesthesiology and Intensive Therapy, Home Mechanical Ventilation Program, Semmelweis University, Budapest, Hungary

Supplementary Table 1. Comparative analysis of baseline parameters for subgroups

|  | Acute patients  (n=15) | Elective patients  (n=15) | *p* value | AHI>30  (n=12) | AHI≤30  (n=18) | *p* value |
| --- | --- | --- | --- | --- | --- | --- |
| ***Anthropometric data*** | | | |  |  |  |
| Age (years) | 52.0 (15.0) | 59.0 (13.0) | p=0.512 | 54.0 (9.0) | 56.0 (21.0) | p=0.819 |
| Male sex | 10 (67.7%) | 12 (80.0%) | p=0.409 | 9 (75.0%) | 13 (72.2%) | p=0.866 |
| BMI (kg/m^2^) | 45.9 (15.2) | 41.5 (12.0) | p=0.067 | 41.2 (11.5) | 44.5 (15.4) | p=0.415 |
| ***Comorbidities*** | | | |  |  |  |
| COPD | 1 (6.7%) | 5 (33.3%) | p=0.069 | 5 (41.7%) | 3 (16.7%) | p=0.129 |
| HTN | 11 (73.3%) | 14 (93.3%) | p=0.142 | 12 (100%) | 13 (72.2%) | p=0.162 |
| CHF | 3 (20.0%) | 4 (26.7%) | p=0.666 | 2 (16.7%) | 5 (27.8%) | p=0.481 |
| PHT | 2 (13.3%) | 2 (13.3%) | p=1.000 | 1 (8.3%) | 3 (16.7%) | p=1.511 |
| CAD | 3 (20.0%) | 4 (26.7%) | p=0.666 | 4 (33.3%) | 3 (16.7%) | p=0.290 |
| DM | 6 (40.0%) | 8 (53.3%) | p=0.464 | 6 (50.0%) | 9 (50.0%) | p=1.000 |
| ***Ventilator settings*** | | | |  |  |  |
| IPAP (cmH_2_O) | 24.0 (7.6) | 21.0 (5.7) | p=0.367 | 23.6 (7.3) | 22.3 (5.7) | p=0.573 |
| EPAP (cmH_2_O) | 11.6 (3.9) | 11.7 (4.8) | p=0.539 | 12.0 (4.3) | 11.2 (4.2) | p=0.172 |
| Respiratory rate (1/min) | 16.3 (3.8) | 15.3 (2.3) | p=0.187 | 15.9 (3.1) | 16.1 (2.5) | p=0.983 |
| Tidal volume (mL) | 629.9 (120.0) | 735.0 (165.5) | p=0.074 | 690.0 (158.8) | 676.3 (182.5) | p=0.662 |
| Supplementary O_2_ (L/min) | 3.0 (2.0) | 1.5 (3.0) | **p=0.033** | 1.8 (3.0) | 3.0 (2.9) | p=0.124 |
| Daily ventilation (hour) | 7.6 (1.7) | 7.2 (3.0) | p=0.571 | 8.2 (3.3) | 7.4 (1.5) | p=0.698 |
| ***Clinical parameters*** | | | |  |  |  |
| AHI (/h) | 9.0 (16) | 53.0 (70.3) | **p<0.001** | 71.1 (58.1) | 9.1 (11.1) | **p<0.001** |
| pH | 7.40 (0.04) | 7.41 (0.04) | p=0.345 | 7.40 (0.03) | 7.41 (0.05) | p=0.723 |
| PaO_2_ (mmHg) | 60.4 (9.1) | 61.0 (22.2) | p=0.838 | 57.5 (19.6) | 61.6 (13.4) | p=0.325 |
| PaCO_2_ (mmHg) | 52.3 (14.4) | 45.0 (11.9) | **p=0.041** | 48.7 (9.5) | 48.2 (18.7) | p=0.755 |
| HCO_3_^-^ mmol/L | 31.9 (5.9) | 27.7 (6.5) | p=0.056 | 29.4 (4.1) | 31.6 (8.7) | p=0.465 |
| FVC% | 61.0 (28.0) | 72.0 (32.0) | p=0.250 | 67.5 (30.0) | 63.0 (34.0) | p=0.950 |
| FEV1% | 54.0 (34.0) | 64.0 (41.0) | p=0.512 | 63.5 (32.0) | 56.0 (38.0) | p=0.632 |
| FEV1/FVC% | 93.0 (13.0) | 93.0 (23.0) | p=0.653 | 95.5 (33.0) | 93.0 (11.0) | p=0.917 |
| PEF% | 53.0 (40.0) | 55.0 (35.0) | p=0.345 | 53.5 (36.0) | 55.5 (30.0) | p=0.950 |
| 6MWT (m) | 220 (152) | 285 (146) | p=0.089 | 259.5 (129) | 244 (279) | p=0.285 |
| ESS (points) | 10 (10) | 10 (7) | p=0.902 | 10 (9) | 9.5 (9) | p=0.573 |
| SRI-SS | 57.7 (25.1) | 58.0 (29.9) | p=0.653 | 56.9 (34.9) | 63.5 (22.3) | p=0.755 |

The Mann-Whitney U test was used for continuous data, and the Chi-square test used for categorical data to compare acute and elective groups. Statistically significant results are highlighted in bold. Abbreviations: AHI: apnea-hypopnea index, BMI: body mass index, COPD: chronic obstructive pulmonary disease, HTN: hypertension, CHF: chronic heart failure, CAD: coronary artery disease, DM: diabetes mellitus, PHT: pulmonary hypertension, IPAP: inspiratory positive airway pressure, EPAP: expiratory positive airway pressure, AHI: apnea hypopnea index, PaO_2_ arterial oxygen pressure, PaCO_2_: arterial carbon dioxide pressure, HCO_3_^-^: bicarbonate, FVC%: percentage of expected forced vital capacity, FEV1%: percentage of expected 1 second forced expiratory volume, FEV1/FVC%: percentage of expected 1 second forced expiratory volume/forced vital capacity, PEF%: percentage of expected peak expiratory flow, ESS: Epworth sleepiness scale, SRI-SS: Severe Respiratory Insufficiency Questionnaire summary score.

Supplementary Table 2.

| Parameter | All patients  Baseline | All patients  6 months | *p* value | Acute patients  Baseline | Acute patients  6 months | p value | Elective patients  Baseline | Elective patients  6 months | *p* value |
| --- | --- | --- | --- | --- | --- | --- | --- | --- | --- |
| BMI (kg/m2) | 43.3 (14.3) | 42.8 (12.4) | p=0.236 | 45.9 (15.2) | 45.2 (10.0) | p=0.061 | 41.5 (12.0) | 40.0 (12.9) | p=0.828 |
| IPAP (cmH2O) | 22.4 (6.4) | 21.9 (6.4) | p=0.066 | 24.0 (7.6) | 22.4 (4.4) | p=0.334 | 21.0 (5.7) | 21.3 (5.5) | p=0.112 |
| EPAP (cmH2O) | 11.7 (4.0) | 11.8 (3.6) | p=0.989 | 11.6 (3.9) | 11.9 (4.4) | p=0.155 | 11.7 (4.8) | 10.1 (5.0) | p=0.158 |
| Respiratory rate (/min) | 16.0 (2.4) | 15.9 (2.5) | p=0.254 | 16.3 (3.8) | 16.0 (4.0) | p=0.496 | 15.3 (2.3) | 15.8 (4.0) | **p=0.041** |
| Tidal volume (mL) | 685 (170) | 699.0 (158.8) | p=0.341 | 629.9 (120.0) | 629.8 (180.6) | p=0.300 | 735.0 (165.5) | 710.0 (198.6) | p=0.514 |
| supplementary O2 (L/min) | 2.0 (3.3) | 2.0 (2.0) | **p=0.005** | 3.0 (2.0) | 2.0 (3.0) | **p=0.018** | 1.5 (3.0) | 0.0 (2.0) | p=0.109 |
| daily ventilation (hour) | 7.5 (2.4) | 7.0 (1.8) | **p<0.001** | 7.6 (1.7) | 7.3 (1.7) | **p=0.013** | 7.2 (3.0) | 6.9 (1.9) | **p=0.016** |
| AHI (/h) | 19.4 (49.4) | 2.8 (2.7) | **p<0.001** | 9.0 (16) | 3.1 (3.7) | **p=0.025** | 53.0 (70.3) | 2.6 (2.4) | **p=0.001** |
| pH | 7.41 (0.04) | 7.41 (0.04) | p=0.556 | 7.40 (0.04) | 7.40 (0.04) | p=0.594 | 7.41 (0.04) | 7.42 (0.04) | p=0.111 |
| PaO_2_ (mmHg) | 60.7 (15.9) | 70.5 (21.1) | **p=0.003** | 60.4 (9.1) | 71.0 (19.8) | **p=0.050** | 61.0 (22.2) | 70.1 (21.7) | **p=0.020** |
| PaCO_2_ (mmHg) | 48.7 (13.9) | 39.8 (21.1) | **p<0.001** | 52.3 (14.4) | 40.5 (15.2) | **p=0.016** | 45.0 (11.9) | 39.3 (7.9) | **p=0.001** |
| HCO_3_^-^ (mmol/L) | 30.4 (7.4) | 25.4 (4.9) | **p<0.001** | 31.9 (5.9) | 26.5 (5.1) | **p=0.008** | 27.7 (6.5) | 25.0 (5.0) | **p=0.007** |
| FVC% | 65.5 (33.0) | 77.0 (31.0) | **p<0.001** | 61.0 (28.0) | 74.0 (24.0) | **p<0.001** | 72.0 (32.0) | 78.0 (36.0) | **p=0.007** |
| FEV1% | 60.5 (36.0) | 68.0 (39.0) | **p<0.001** | 54.0 (34.0) | 68.0 (39.0) | **p=0.001** | 64.0 (41.0) | 67.0 (41.0) | **p=0.012** |
| FEV1%/FVC% | 93.0 (18.0) | 94.0 (19.0) | p=0.974 | 93.0 (13.0) | 97.0 (13.0) | p=0.776 | 93.0 (23.0) | 91.0 (20,0( | p=0.875 |
| PEF% | 54.5 (31) | 62.0 (29.5) | **p=0.012** | 53.0 (40.0) | 63.0 (34.0) | p=0.069 | 55.0 (35.0) | 59.5 (29.3) | p=0.052 |
| 6MWT (m) | 252 (174) | 325.0 (182.0) | **p<0.001** | 220 (152) | 305.0 (245.0) | **p<0.001** | 285 (146) | 350.0 (131.0) | **p=0.001** |
| ESS (points) | 10 (9) | 3 (3) | **p<0.001** | 10 (10) | 2.0 (3.0) | **p<0.001** | 10 (7) | 4.0 (5.0) | **p<0.001** |
| SRI-SS | 57.9 (27.0) | 76.8 (24.1) | **p<0.001** | 57.7 (25.1) | 85.1 (22.6) | **p=0.003** | 58.0 (29.9) | 75.5 (35.1) | **p<0.001** |

Abbreviations: BMI: body mass index, IPAP: inspiratory positive airway pressure, EPAP: expiratory positive airway pressure, AHI: apnea hypopnea index, PaO_2_ arterial oxygen pressure, PaCO_2_: arterial carbon dioxide pressure, HCO_3_: bicarbonate, FVC%: percentage of expected forced vital capacity, FEV1%: percentage of expected 1 second forced expiratory volume, FEV1/FVC%: percentage of expected 1 second forced expiratory volume/forced vital capacity, PEF%: percentage of expected peak expiratory flow, ESS: Epworth sleepiness scale, SRI-SS: Severe Respiratory Insufficiency Questionnaire summary score, 6MWT: 6 minute walking test.

Supplementary Table 3. Baseline biomarker values for all patients and different subgroups.

|  | All patients  (n=30) | Acute patients  (n=15) | Elective patients  (n=15) | *p* value | AHI > 30 patients  (n=12) | AHI ≤ 30 patients (n=18) | *p* value |
| --- | --- | --- | --- | --- | --- | --- | --- |
| ***Hematological*** | | | | | | | |
| RBC (T/L) | 5.2 (1.0) | 5.2 (1.3) | 5.3 (0.8) | p=0.486 | 5.3 (1.0) | 5.0 (1.1) | p=0.415 |
| RDW (%) | 15.1 (3.3) | 15.4 (3.9) | 14.7 (2.1) | p=0.148 | 14.7 (1.4) | 16.1 (5.2) | **p=0.048** |
| Hb (g/L) | 147.5 (33.0) | 142.0 (50.0) | 155.0 (20.0) | **p=0.045** | 155.5 (26.0) | 145.0 (38.0) | **p=0.035** |
| Hct (L/L) | 0.46 (0.10) | 0.46 (0.13) | 0.45 (0.08) | p=0.486 | 0.47 (0.08) | 0.43 (0.12) | p=0.249 |
| PLT (G/L) | 246.0 (129.0) | 246.0 (158.0) | 246.0 (92.0) | p=0.512 | 249.5 (158.0) | 235.0 (160.0) | p=0.950 |
| Ferritin (umol/L) | 13.3 (7.2) | 13.1 (7.6) | 13.4 (7.0) | p=0.806 | 15.0 (8.2) | 11.2 (6.6) | **p=0.025** |
| TIBC (umol/L) | 70.7 (29.7) | 65.0 (21.6) | 80.3 (28.4) | p=0.067 | 78.2 (21.3) | 64.5 (33.4) | p=0.065 |
| ***Nutrition*** | | | | | | | |
| Total protein (g/L) | 67.4 (7.5) | 64.6 (12.2) | 69.5 (4.7) | **p=0.033** | 70.4 (6.9) | 65.0 (9.3) | **p<0.001** |
| Albumin (g/L) | 38.7 (7.1) | 34.0 (6.2) | 40.8 (3.1) | **p<0.001** | 40.3 (3.9) | 35.0 (8.3) | **p<0.001** |
| TG (mmol/L) | 2.0 (1.1) | 1.7 (0.9) | 2.2 (1.0) | p=0.202 | 2.1 (1.4) | 1.9 (1.0) | p=0.632 |
| Cholesterol (mmol/L) | 4.9 (1.4) | 4.2 (1.1) | 5.3 (1.5) | **p=0.009** | 5.1 (2.5) | 4.6 (1.0) | p=0.267 |
| Transferrin (g/L) | 2.90 (1.06) | 2.61 (0.86) | 3.20 (1.13) | p=0.098 | 3.13 (0.85) | 2.66 (1.33) | p=0.104 |
| Na (mmol/L) | 140.0 (5.0) | 139.0 (5.0) | 140.0 (5.0) | p=0.686 | 139.0 (6.0) | 140.0 (4.0) | p=0.415 |
| K (mmol/L) | 4.4 (0.7) | 4.4 (0.4) | 4.2 (1.1) | p=0.345 | 4.2 (1.0) | 4.4 (0.5) | p=0.632 |
| Mg (mmol/L) | 0.81 (0.51) | 0.82 (0.14) | 0.78 (0.18) | p=0.345 | 0.80 (0.18) | 0.81 (0.13) | p=0.723 |
| ***Muscle*** | | | | | | | |
| Creatinine (umol/L) | 69.5 (21.0) | 67.0 (28.0) | 75.0 (23.0) | p=0.412 | 74.0 (20.0) | 67.5 (28.0) | p=0.368 |
| BUN (mmol/L) | 6.2 (3.4) | 5.5 (4.9) | 7.0 (2.4) | p=0.126 | 6.1 (2.4) | 6.1 (4.8) | p=0.249 |
| CK (U/L) | 76.0 (58.0) | 60.0 (69.0) | 99.0 (53.0) | p=0.137 | 101.0 (68.0) | 52.5 (100.0) | **p=0.010** |
| Total calcium (mmol/L) | 2.4 (0.2) | 2.3 (0.2) | 2.4 (0.2) | **p<0.001** | 2.4 (0.2) | 2.3 (0.2) | **p=0.013** |
| Phosphate (mmol/L) | 1.11 (0.29) | 1.2 (0.4) | 1.1 (0.2) | p=0.345 | 1.1 (0.4) | 1.1 (0.3) | p=1.000 |
| ***Cardiovascular*** | | | | | | | |
| NT-proBNP (pg/mL) | 177.3 (483.6) | 181.8 (782.8) | 65.8 (398.0) | p=0.389 | 41.5 (370.2) | 199.9 (891.3) | p=0.065 |
| HS troponin I (ng/L) | 5.1 (24.8) | 8.5 (44.5) | 4.6 (21.2) | p=0.161 | 4.6 (17.3) | 8.3 (40.6) | p=0.368 |
| CKMB (U/L) | 12.0 (9.0) | 11.0 (8.0) | 13.0 (3.0) | p=0.202 | 13.0 (4.0) | 11.0 (8.0) | p=0.104 |
| HDL (mmol/L) | 1.0 (0.4) | 0.9 (0.2) | 1.2 (0.3) | **p<0.001** | 1.1 (0.4) | 0.9 (0.4) | p=0.215 |
| LDL (mmol/L) | 3.1 (1.1) | 2.9 (0.8) | 3.3 (1.0) | p=0.061 | 3.2 (2.1) | 3.1 (0.8) | p=0.491 |
| Lipoprotein (g/L) | 0.11 (0.51) | 0.12 (0.36) | 0.10 (0.76) | p=0.715 | 0.42 (0.78) | 0.09 (0.22) | p=0.188 |
| ***Inflammation and fatigue*** | | | | | | | |
| WBC (G/L) | 7.9 (2.9) | 7.5 (5.0) | 8.6 (2.7) | p=0.106 | 8.8 (2.6) | 7.6 (3.3) | p=0.124 |
| CRP (mg/L) | 10.5 (21.7) | 15.5 (22.6) | 7.2 (11.2) | p=0.106 | 5.6 (10.4) | 14.7 (20.7) | p=0.106 |
| IL-6 (pg/mL) | 5.8 (10.3) | 9.7 (12.4) | 2.2 (5.0) | **p<0.001** | 4.7 (6.8) | 8.1 (14.4) | p=0.065 |
| IL-1ra | 1151.4 (1921.7) | 2128.7 (4080.8) | 650.8 (593.8) | **p<0.001** | 977.0 (869.5) | 1621.8 (2937.3) | p=0.059 |
| sTNF RII | 3756.6 (1788.4) | 4950.3 (874.9) | 3422.3 (945.0) | **p<0.001** | 3426.0 (470.8) | 4826.3 (1716.8) | **p=0.035** |
| Activin A (pg/mL) | 340.9 (118.9) | 380.7 (495.4) | 326.6 (117.4) | **p=0.012** | 321.6 (116.0) | 371.3 (236.8) | p=0.068 |
| ApoA1 (g/L) | 1.36 (0.38) | 1.05 (0.21) | 1.40 (0.35) | **p<0.001** | 1.34 (0.25) | 1.17 (0.48) | p=0.200 |
| ApoB (g/L) | 0.87 (0.35) | 0.97 (0.38) | 0.96 (0.40) | p=1.000 | 0.97 (0.41) | 0.98 (0.36) | p=0.662 |

Baseline biomarker values for all patients and subgroups. Mann-Whitney *p* values comparing subgroups are listed. Significant data are marked with bold font. Abbreviations: ApoA1: apolipoprotein A1, ApoB: apolipoprotein B, BUN: blood urea nitrogen, CRP: C-reactive protein, CK: creatine kinase, CKMB: creatine kinase myoglobin-binding, Hb: hemoglobin, Hct: hematocrit, HDL: high-density lipoprotein, HS troponin I: high sensitivity troponin I, IL-6: interleukin-6, IL-1ra: interleukin-1 receptor antagonist, LDL: low-density lipoprotein, NT-proBNP: N-terminal pro-brain natriuretic peptide, RBC: red blood cell, RDW: red cell distribution width, sTNF RII: soluble tumor necrosis factor receptor II, TG: triglyceride, TIBC: total iron binding capacity, WBC: white blood cell
